# Temperature Variation and Heat Wave and Cold Spell Impacts on Years of Life Lost Among the Urban Poor Population of Nairobi, Kenya

**DOI:** 10.3390/ijerph120302735

**Published:** 2015-03-02

**Authors:** Thaddaeus Egondi, Catherine Kyobutungi, Joacim Rocklöv

**Affiliations:** 1 African Population and Health Research Center, Nairobi, P.O. Box 10787-00100, Kenya; E-Mail: ckyobutungi@aphrc.org; 2 Department of Public Health and Clinical Medicine, Epidemiology and Global Health, Umeå University, Umeå, SE–901-87, Sweden; E-Mail: joacim.rocklov@umu.se

**Keywords:** cold-related mortality, heat-related mortality, heat wave, cold spell, temperature

## Abstract

Weather extremes are associated with adverse health outcomes, including mortality. Studies have investigated the mortality risk of temperature in terms of excess mortality, however, this risk estimate may not be appealing to policy makers assessing the benefits expected for any interventions to be adopted. To provide further evidence of the burden of extreme temperatures, we analyzed the effect of temperature on years of life lost (YLL) due to all-cause mortality among the population in two urban informal settlements. YLL was generated based on the life expectancy of the population during the study period by applying a survival analysis approach. Association between daily maximum temperature and YLL was assessed using a distributed lag nonlinear model. In addition, cold spell and heat wave effects, as defined according to different percentiles, were investigated. The exposure-response curve between temperature and YLL was J-shaped, with the minimum mortality temperature (MMT) of 26 °C. An average temperature of 21 °C compared to the MMT was associated with an increase of 27.4 YLL per day (95% CI, 2.7–52.0 years). However, there was no additional effect for extended periods of cold spells, nor did we find significant associations between YLL to heat or heat waves. Overall, increased YLL from all-causes were associated with cold spells indicating the need for initiating measure for reducing health burdens.

## 1. Introduction

Extreme weather has been associated with excess morbidity and mortality in many different regions, consequently, there is a growing interest in understanding the relationship between weather variations and health and how this will be manifest as climate change takes hold and extreme weather events become more common [1,2]. Extreme temperatures are known to exacerbate certain medical problems, like heart disease, and excess deaths have been observed during periods of extreme temperatures in different cities around the world [3]. Generally, most epidemiological studies have shown short-term effects of ambient temperature on overall mortality, with a relationship of the increased risks in cold and hot weather [4,5]. The effects of extreme temperatures may be delayed to last for some days [6,7]. Some studies have investigated extended periods of extreme temperatures, known as cold spells and heat waves, and found these to be associated with peaks in mortality [8,9].

However, even moderate deviations from temperature norms can pose risks to human. Temperature variability is a key factor explaining differences in temperature-related mortalities across regions [10,11]. Current findings show that when the temperature changes considerably over the course of a few days, even without reaching extremes, it is harmful for vulnerable populations, especially those with existing health problems [12]. Evidence of a temperature-mortality relationship exists also for sub-tropical countries that experience moderate temperature variations [13,14,15]. In addition, there is evidence of adaptation to temperature extremes [10,16,17] as cold and heat effects have different thresholds for increased risk onset for different regions.

Most of these previous studies have investigated the weather-related mortality by assessing whether there is evidence of excess mortality during extreme weather [18,19,20]. However, the relative risks are believed to be heavily driven by short-term mortality displacement. It has been argued that it would be more informative to assess mortality impact related to temperature exposure using estimates that takes into account the life expectancy [21]. Depending on whether most temperature-related mortality occur among people with shorter or longer life expectancy, the expected magnitude of years of life lost (YLL) is expected to differ [22]. Summarizing temperature-mortality association in terms of attributable risk on estimates such as YLL offers adequate information on the actual impact of exposure to temperature variations [23,24]. YLL is a measure of disease burden that uses the life expectancy at death. It gives more weight to deaths among younger people compared with the traditional measure of relative mortality risk that weights all deaths equally [25].

Very little is known on weather-mortality relationship among the urban poor population in (sub-Saharan) Africa. One previous study among the urban poor population in Nairobi evaluated the effect of temperature on all-cause mortality in two informal settlements in terms of mortality risk [13]. It was found that both low and high temperatures were associated with excess mortality. This paper extends the investigation of weather-related health impacts by examining the association of temperature on YLL using data from two informal settlements in Nairobi. In addition, we also investigated whether there are additional cold spell and heat wave effects on YLL. Besides contributing to evidence on weather-related mortality, the paper provides information on the burden of weather extremes in terms of YLL among a population faced with multiple health and socio-economic problems rendering it vulnerable to environmental exposures.

## 2. Methods

### 2.1. Study Area

The study was based on data from Nairobi Urban Health and Demographic Surveillance System (NUHDSS) run in the two slums of Korogocho and Viwandani in Nairobi, Kenya since 2003. Approximately, a population of 66,000 people were under surveillance in the two study sites by the year 2012. Nairobi is the capital of Kenya, located at 1°16*'* Latitude South and 36°48*'* Longitude East at an altitude of 1700 m. Although the city of Nairobi is situated close to the Equator, it experiences an equable climate as opposed to tropical climate. The city experiences a short rainy period in November/December and a heavy rainy season from March till the beginning of June. The city has a population of about 3.1 million, with more than a half of the population residing in informal settlements [26].

### 2.2. Data

Daily mortality data from 1 January 2003 to 31 December 2012 (the whole period for which data are available) were obtained from the NUHDSS database. All deaths among residents from the two study areas and information on movements in-and-out of the study areas were recorded. The data included date of start event (birth, enumeration or in-migration), date of last event (death, outmigration or exit), sex, age, and cause of death. However, for this analysis we considered all-cause mortality. The data were used to generate age-sex specific life expectancies to estimate the YLL for each death by matching their age and sex. The daily YLL are the total YLL for all deaths that occurred on the same day. The calculation of YLL for each death $(YLL_{i})\;$and subsequently for each day $(YLL_{t})\;$can be summarized by the following two expressions:

(1) $$YLL_{i}=LE_{r}-(A-\left(ALB+0.5\right))$$

(2) $$YLL_{t}=\;\overset{n}{\underset{i=1}{\sum }}YLL_{i}$$

where A is the actual age at death for individual *i*, $LE_{r}$ is the remaining life expectancy at age *A* and *ALB* is the age lower bound from the life table. The letter *t* denotes day of the year and *n* is the number of deaths on that particular day of the year. The expression is modified from the general formula for group YLL [27] to calculate individual YLL and account for the fraction of a year lived. The applied calculation of YLL does not incorporate age discounting, which adjusts for social preferences for the age at which death occurred. To illustrate first expression, we consider an individual who died with an actual age of 75.86 and the life expectancy at age 75 is 9.85—the YLL for this individual is obtained as 9.85 ‒ (75.86 ‒ (75 + 0.5)) = 9.85 ‒ 0.36 = 9.49.

Daily weather data were obtained from the National Oceanic and Atmospheric Administration (NOAA) website (http://www.ncdc.noaa.gov/data-access/land-based-station-data/land-based-datasets) for the Moi Airbase Eastleigh weather station for the study period of 2003 to 2012. The weather station is located in between the two study sites (4 kilometres from each study area). We obtained daily values of maximum, minimum, and mean temperatures. When daily weather data were missing (22% of the days) for the Moi Airbase station, data from Jomo Kenyatta International Airport (JKIA) were used. The temperature data from the two stations were found to be highly correlated with a correlation coefficient of 0.92 (92%). The two weather stations are 9 kilometres apart. The daily maximum temperature was used for the analysis because it is used for defining the cold and hot days [28].

### 2.3. Definitions of Temperature Extremes

Due to a lack of standard definitions of a cold spell or heat wave, we used percentiles as suggested in the literature to define thresholds for extreme cold and heat over a period of consecutive days [29]. These definitions describe heat wave and cold spells relative to the usual weather in the area and relative to normal temperatures for the season. This implies that temperature people from a hotter climate consider normal can be termed a heat wave in a cooler area if they are outside the normal climate pattern for that area [30,31]. We considered a combination of different intensities measured by percentiles and duration measured by the days of sustained temperature extreme to define cold spells and heat waves [32,33]. The indicator variables for cold or heat were defined according to different percentiles of the temperature distributions expressed as:

(3) $$I_{C}=\{\begin{array}{c} 1\;if\;temperature<\tau_{c}\\ 0\;otherwise \end{array}\;\text{and}\;I_{H}=\{\begin{array}{c} 1\;if\;temperature>\tau_{H}\\ 0\;otherwise \end{array}$$

where $I_{C}$ and $I_{H}$ represent indicators for cold spell and heat wave days, respectively. The different percentiles of 90th, 95th and 98th were used for different intensities of heat waves and 10th, 5th and 2nd percentiles for different intensities of cold spells. Therefore, cold spell and heat wave were defined as indicators for at least two days of consecutive days with temperatures below or above the identified temperature thresholds for cold and heat. The sustained heat wave or cold spells of at least 2 days with extreme temperature were considered for analysis. As an example for at least 2 days of sustained cold spell, if July 5th and 6th were days with maximum temperatures below the cold threshold, then July 6th would have a value of “1” for cold spell while July 5th would have a value of 0. A similar approach was used to define values for heat wave days.

### 2.4. Data Analysis

The analysis followed a scheme for evaluating the effect of cold and heat waves proposed in the literature [32,33]. The methodology decomposes the effects of cold and heat into main and added effects. The general model is represented as:

(4) $$E\left(Y_{t}\right)=\mu_{t}=\;\alpha +s\left(temp,df\right)+s\left(time,df\right)+I_{C}+I_{H}+\varepsilon_{t}$$

The outcome, $Y_{i}$ is the daily total YLL for day *i* assumed to follow a normal distribution. The smoothing function*, s(.)* represents the nonlinear relationship of main temperature and time trend which accounts for both long term as well as seasonal variation. The term *df* represents degrees of freedom while $I_{C}$ and $I_{H}$ represent the indicators for cold spell and heat wave as defined above. The analysis was performed in two parts: first, the general association of temperature and YLL was estimated; secondly, the additional effect of cold spells and heat waves on YLL was assessed.

### 2.5. General Association of Temperature and YLL

We used a distributed lag nonlinear model to estimate the association between daily maximum temperature and daily total YLL. The approach has been widely used to model the association of environmental exposure and health outcomes [32,34]. This model allows the main effect to vary along both temperature and lag dimensions. The relationship in the temperature space was captured using a natural spline with two *df*. To capture the delayed effects of temperature, we considered a maximum lag of 14 days between exposure and death then evaluated the sensitivity of the results using a maximum lag of 21 days. The lag effect was modelled using a natural spline with three *df*. The choice of *df* for temperature and lag splines was made after comparing AICs for different combinations of *df* for both terms. We adjusted for both long-term trends and seasonality in the YLL estimates using a natural cubic spline with a total of 60 *df* corresponding to six *df* per year based on sensitivity from earlier study [13]. The general distribution of expected YLL and daily maximum temperature was plotted with corresponding 95% confidence intervals (CIs).

### 2.6. Effects of Cold Spells and Heat Waves

First, the lag effect of the cold spells and heat waves were explored and displayed graphically considering a maximum lag of 14 days and check for the sensitivity using a maximum lag of 21 days. The different lag effects were not adjusted for each other; each lag was fitted in the model one at a time. The cold spell and heat wave indicators were included in the above described general model to assess the effect of cold and hot temperatures. The main effect of cold (or heat) was obtained from the model by predicting expected YLL between the median temperatures among cold (or heat) wave days *versus* the minimum mortality temperature (MMT). The MMT was obtained from the predictions of the overall cumulative exposure-response association. The heat wave and cold spell indicator variables were included in the model to estimate the additional effect of heat wave and cold spell. Therefore, those estimates compared YLL on extreme temperature days with non-extreme days.

## 3. Results

### 3.1. Characteristics of Temperature, Daily Mortality and YLL

The average daily maximum temperature in the study area was 25.8 °C (median of 26.0 °C), with a minimum of 15.0 °C and a maximum of 38.2 °C. The inter-quartile range of the daily maximum temperature was 24.1–27.6 °C. Summary statistics for daily mortality and YLL for a period of 2003–2012 are provided in Table 1. A total of 4671 deaths were recorded in the NUHDSS during the study period, with an average of 1.3 deaths per day. There was not much variation in the daily average number of deaths between gender and by age group. However, there were more deaths among males (56.8%) compared to females (43.2%). There were 56.6 YLL per day due to all causes of death and a total of 206,712 YLL over the study period.

**Table 1:** Descriptive summary statistics for daily deaths and YLL in NUHDSS by gender and age group (2003–2012).

	Daily Average Deaths (SD)	Total Deaths	Daily Average YLL (SD)	Total YLL
Sex/Gender				
Male	0.7 (1.3)	2651	31.9 (56.7)	116,349.4
Female	0.6 (0.9)	2020	24.7 (42.6)	90,362.9
Age group				
0–5 years	0.4 (0.7)	1487	25.6 (44.1)	93,460.2
5–15 years	0.0 (0.2)	146	2.4 (12.0)	8601.5
15–25 years	0.1 (0.4)	415	5.5 (19.4)	20,049.3
25–50 years	0.5 (1.2)	1966	19.8 (19.8)	72,263.4
50+ years	0.2 (0.5)	657	3.4 (9.5)	12,337.9
**Overall**	**1.3 (1.9)**	**4671**	**56.6 (82.0)**	**206,712.3**

**Figure 1:**
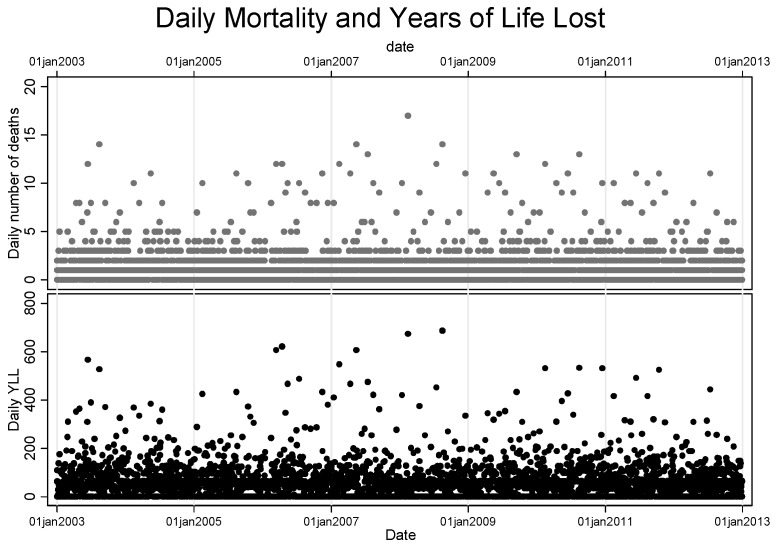
Distribution of deaths and YLL the over study period.

Figure 1 shows scatter plots for the distribution of both the daily mortality and YLL over time for the entire study period. The plot shows a similar trend pattern for both measures but with a discernible difference in the magnitude.

### 3.2. Association of Temperature and YLL

**Figure 2:**
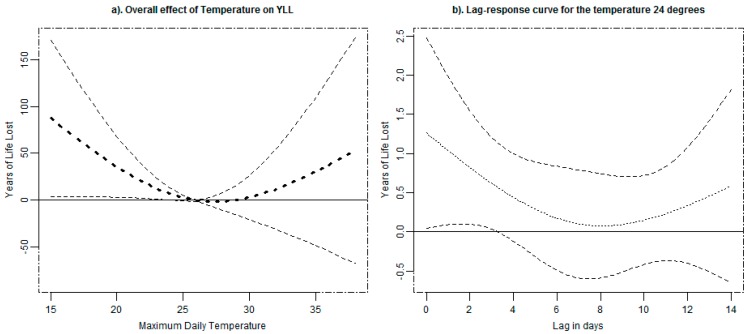
The overall effects of temperature on YLL (**a**) and the corresponding lag-response for temperature of 24 °C (**b**) due of all-cause mortality in NUHDSS with 95% confidence intervals.

The general association of temperature and YLL is illustrated in Figure 2a which represents the cumulative effect over 14 days. The exposure–response curve between daily temperature and YLL is J-shaped, with the lowest YLL between 24 and 30 °C. The predictions from the exposure-response curve indicate 26 °C as the MMT, and a corresponding minimum mortality percentile (MMP) of 60%. The plot indicates the existence of an association between cold temperatures and YLL. For a constant exposure to an average temperature of 21 °C (5th percentile) for 14 days, the YLL will increase by 27.4 YLL in a given day (95% CI, 2.7–52.0 years) for a change of temperature from 26 °C. Considering the association between heat and YLL, there was an increase of 3.3 YLL in a given day for a constant exposure to an average temperature of 30 °C for 14 days, a change from the reference temperature of 26 °C (95% CI, −19.7–26.4 years). The cumulative association of temperature and YLL, thus, did not show a significant heat effect in the study area. As part of sensitivity we considered the maximum lag of 21 days and obtained non-significant results for both cold and heat effects *i.e.*, 22.8 (−7.9–53.5) and 12.3 (−16.3–40.9) respectively. The lag effect specific to 25th percentile temperature of 24 °C is shown in Figure 2b. The graph is generated by plotting YLL corresponding for the temperature of 24 °C for different lags. The lag curve in the plot represents an increase in YLL for each future day following exposure to a temperature of 24 °C. The figure shows the delayed effect for average temperature of 24 °C which diminishes after six days of exposure.

### 3.3. Effects of Heat Waves and Cold Spells

**Figure 3:**
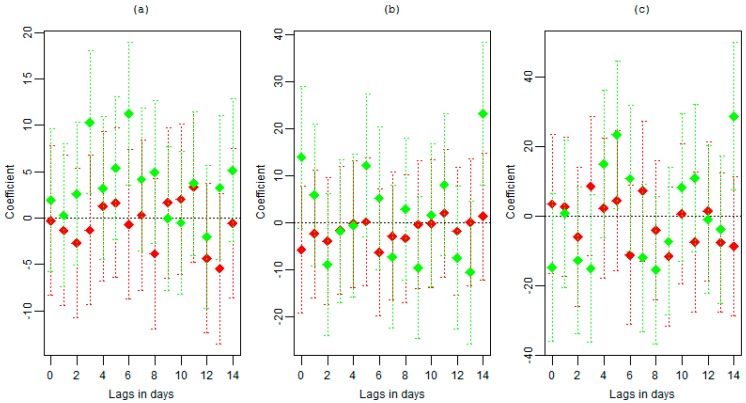
The delayed associations of cold spell (green) and heat wave (red) of at least two days heat wave or cold spell on years of life for different definitions: (**a**) <10th and >90th percentiles, (**b**) <5th and >95th percentiles and (**c**) <2nd and >98th percentiles.

Figure 3 shows the delayed associations of cold spell and heat wave on YLL due for different intensities in addition to the association presented in Figure 2 up to lag of 14 days. The plots show no clear pattern for both cold spells and heat waves. However, a cold spell signal that is significant at different lags and for different intensities is visible.

Thresholds based on different percentiles and number of consecutive days of cold spell/heat wave with corresponding effect are given in Table 2. The association of cold spell defined as the 10th percentile representing 22.4 °C showed significance at lag 3 and 6 days. Cold spells defined as the 5th percentile representing 21.1 °C showed significance at 14 and some effect at lag 5. The high intensity cold wave defined by 2nd percentile showed similar effect as cold spells defined by the 5th percentile with significance at lag 5 and 14.

The lag effect for the cold spells seems to last up to 14 days since there is no significance observed after 14 days up to 21 days (data not shown). However, when considering the maximum lag up to 21 days, the heat wave effect seems to be present at long lags of 18–19 days. The heat wave defined by 98th percentile showed significant effect at lag 18 and 19. These graphical results of exploring the lag effect were based on separate models for different lags.

**Table 2:** Heat wave and cold spell temperature thresholds and number of consecutive days with corresponding effect on YLL.

	Threshold (°C)	No. of Days	Main Effect			Added Effect		
			YLL	95% CI		YLL	95% CI	
*Cold Spell Intensities*								
≤2nd percentile	20.0	24	56.7	4.4	109.1	−6.2	−42.8	30.4
≤5th percentile	21.1	67	35.8	2.3	69.2	−0.4	−24.2	23.5
≤10th percentile	22.4	169	26.1	0.6	51.6	1.9	−14.8	18.5
*Heat Wave Intensities*								
≥98th percentile	29.0	23	3.4	−20.7	27.5	−0.6	−37.5	36.2
≥95th percentile	29.6	88	1.3	−25.5	28.2	−1.6	−16.5	13.3
≥90th percentile	30.4	221	8.1	−28.0	44.2	7.5	−12.9	27.9

Table 2 shows average YLL associated with the main and added effects of heat waves and cold spells in those days matching the different definitions cumulatively over 14 lags. The results show the existence of a cold spell signal. However, as observed from the lag plots and the general temperature-YLL response, there is no significant association between heat wave and all-cause mortality observed in the study area.

A significant association between low temperatures and YLL was observed and increased with the intensity (10th, 5th and 2nd percentiles) computed relative the median temperature during the cold spell days. The median temperature for days with temperatures below 2nd percentile (20.0 °C) of 18.0 °C was associated with 53.2 YLL (95% CI: 1.3 to 105.0). The median temperature of 21.0 °C for least strict definition of days defined by temperature below 10th was associated with a loss of 23.7 years (95% CI: 0.6 to 51.6). These effects were estimated relative to the minimum mortality temperature of 26 °C corresponding to minimum mortality percentile of 60th. Upon increasing the lag effect to a maximum of 21 days, a similar result as that observed for exposure-response association of no significant effect for both cold and heat. However, there was an increase in the heat wave estimates with no much change for cold spells. After observing this no significant addition effect, we fitted the model without the cold and heat indicators. The reduced model produced small changes in the main cold spell and heat wave effect estimates.

## 4. Discussion

The study characterizes the health burden associated with temperature variations in an urban informal residential area in Nairobi, Kenya. The impacts of weather and climate on population health in this region are to date under-researched. The study brought forward estimates on how temperature and heat and cold waves impact on population level YLLs. The existence of cold spell and heat wave effects were explored using a newly described approach that has been developed for studies of mortality counts [32,33], but here expressed in terms of YLL. The study took full advantage of the longitudinal cohort design provided by the NUHDSS and its mortality data to enable this health risk assessments of temperature extremes among the socially deprived population. In this study, we considered the effect of temperature on YLL from all-cause mortality. We found that the association between temperature and YLL was a J-shaped curve, with a significant increase of YLL associated with cold temperatures. We observed a significant cold spell association with YLL for the three different definitions ranging from an average of 26 to 57 YLL per day of cold spell. We found that the effects of temperature on the YLL were well explained by the overall relationship of temperature to mortality, and that heat or cold waves did not appear to result in additional mortality effects.

The majority of previous studies have examined the relative risk of temperature-related mortality [4,35], and only a few have considered YLL [22,23,36]. YLL is an informative measure for assessing the health impacts from weather compared to mortality risk as it accounts for deaths at different ages by gender. It also accounts for mortality displacement by definition in addition to providing information on the preventable loss of life years due to the exposure [25,37]. Understanding the impact of temperature exposure on YLL is helpful in evaluating the health risks for other exposures in a population such as slum residents who are faced with multiple health risk stressors.

Cold weather is associated with a variety of involuntary responses in humans, including peripheral vasoconstriction (contraction of skin blood vessels), shivering, and increased blood pressure and heart rate. Therefore, for patients with heart disease, exposure to cold may cause a decrease in coronary blood flow leading to coronary spasms, chest pains, and even myocardial infarction [38,39,40]. Cold weather interferes with lung mechanisms and available biological information is sufficient to make plausible hypothesis that exposure to cold is a risk factor for pneumonia in all ages [41]. Exposure to cold weather is also often associated with use of fires among slum residents to generate warmth and correspondingly increase exposure to air pollution causing both higher indoor and outdoor air pollution levels. On the contrary, exposure to heat has been found to induce physiological changes such as an increases in blood viscosity and cardiac output leading to dehydration and hypotension [42,43]. In fact, exposure to extreme temperatures in general (both cold and heat) can act as a trigger for cardiovascular events due to changes in blood pressure, blood viscosity, blood cholesterol, and heart rate [44,45,46].

This study showed that an effect of cold temperatures on YLL was observed and was limited to the first five days after exposure. Cold-related mortality has been established before in different regions [14,15,16,47]. This study found no significant added impact of neither cold spells nor heat waves in general. This is similar to many other studies [32,36] and was expected in this kind of setting with a small temperature range. The observed effect of cold is consistent with results for a multi-country study that reports more than 70% of temperature related mortality attributable to cold across countries and minimum mortality percentile is similar to that reported for sub-tropical countries to be around 60th percentile [48].

It is important to create greater awareness of the dangers of extreme temperatures to inform the public about how to minimize their risks. However, there is need for individual level studies to establish vulnerable groups and help in designing adaptive strategies [44]. The information on the delayed effect of temperature exposure and YLL is vital for developing response plans for extreme temperature events [18,36]. However, it is likely that basic improvements of housing conditions, and better sources of heating in the housing particularly in the slum areas of Nairobi would mitigate risks related to cold exposures. The fact that the slum population is deprived of basic amenities, they may have no alternatives, even if they are aware of the dangers related to exposure to cold temperatures. Exposed to multiple environmental stressors together with poor housing facilities [49], the slum residents are less likely to acclimatize to temperature variations. The association of poor or old buildings, manual work, low socio-economic status and temperature variation [50] implying that the slum population is a more vulnerable group. These characteristics hinder this kind of deprived population from adapting to weather variations.

The study contributes to a better understanding of the burden of temperature variations in a location with a smaller temperature range among the disadvantaged population in Africa. The study uses a new analytical approach which offers several advantages. Among those: the use of distributed non-linear functions gives assurance that the main effect is adequately accounted for; the analysis takes into consideration different definitions of cold spell and heat wave intensities; the study allows a more accurate estimation of temperature effects by making a distinction between effects from independent daily temperatures and from the duration of prolonged extreme temperatures. However, we acknowledge some study limitations. First, the data were available for only two slum areas, which makes our results hard to generalize to other communities in Nairobi and beyond. Second, temperature measurements were obtained from a monitoring station located outside the study area, which may not represent well the actual individual exposures, creating a potential misclassification of exposure. However, we consider that the fact that the slum population is not well protected from low and high temperatures by housing standards counteracts this exposure bias. We also acknowledge the small population under study that resulted in a small number deaths per day which might affect the power of the study.

## 5. Conclusions

To conclude, this study shows evidence that exposure to ambient temperature variation is associated with YLL due all causes of death in Nairobi; specifically, cold temperatures appear more harmful. This is contrary to the general believe that Nairobi is a cool city compared to other African locations. Understanding of people’s perceptions and behavior towards weather extremes is required for better targeted awareness campaigns to reduce the health burden from temperature exposure. The findings also point to the need to think of environmental exposures in an effort to reduce disease burden among the urban poor population.

## References

[1] Huang C., Barnett A., Wang X., Vaneckova P., FitzGerald G., Tong S. (2011). Projecting future heat-related mortality under climate change scenarios: A systematic review. Environ Health Perspect..

[2] Luber G., McGeehin M. (2008). Climate change and extreme heat events. Am. J. Prev. Med..

[3] Kovats R.S., Hajat S. (2008). Heat stress and public health: A critical review. Ann. Rev. Public Health.

[4] Hajat S., Kosatky T. (2010). Heat-related mortality: A review and exploration of heterogeneity. J. Epidemiol. Community Health.

[5] O’Neill M., Ebi K. (2009). Temperature extremes and health: Impacts of climate variability and change in the United States. J. Occup. Environ. Med..

[6] Goldberg M., Gasparrini A., Armstrong B., Valois M. (2011). The short-term influence of temperature on daily mortality in the temperate climate of Montreal, Canada. Environ. Res..

[7] Rocklöv J., Forsberg B. (2008). The effect of temperature on mortality in Stockholm 1998–2003: A study of lag structures and heatwave effects. Scand. J. Public Health.

[8] Le Tertre A., Lefranc A., Eilstein D., Declercq C., Medina S., Blanchard M., Chardon B., Fabre P., Filleul L., Jusot J. (2003). Impact of the 2003 heatwave on all-cause mortality in 9 French cities. Epidemiology.

[9] Revich B., Shaposhnikov D. (2008). Excess mortality during heat waves and cold spells in Moscow, Russia. Occup. Environ. Med..

[10] Braga A.L., Zanobetti A., Schwartz J. (2001). The time course of weather-related deaths. Epidemiology.

[11] Medina-Ramón M., Schwartz J. (2007). Temperature, temperature extremes, and mortality: A study of acclimatisation and effect modification in 50 US cities. Occup. Environ. Med..

[12] Zanobetti A., O'Neill M.S., Gronlund C.J., Schwartz J.D. (2012). Summer temperature variability and long-term survival among elderly people with chronic disease. Proceed. Natl. Acad. Sci..

[13] Egondi T., Kyobutungi C., Kovats S., Muindi K., Ettarh R., Rocklöv J. (2012). Time-series analysis of weather and mortality patterns in Nairobi's informal settlements. Global Health Action.

[14] Gouveia N., Hajat S., Armstrong B. (2003). Socioeconomic differentials in the temperature-mortality relationship in São Paulo, Brazil. Int. J. Epidemiol..

[15] Wu W., Xiao Y., Li G., Zeng W., Lin H., Rutherford S., Xu Y., Luo Y., Xu X., Chu C. (2013). Temperature–mortality relationship in four subtropical Chinese cities: A time-series study using a distributed lag non-linear model. Sci. Total Environ..

[16] Keatinge W.R., Donaldson G.C., Cordioli E., Martinelli M., Kunst A.E., Mackenbach J.P., Nayha S., Vuori I. (2000). Heat related mortality in warm and cold regions of Europe: Observational study. BMJ (Clin. Res. Ed.).

[17] Pope C.A.I. (2007). Mortality effects of longer term exposures to fine particulate air pollution: Review of recent epidemiological evidence. Inhal. Toxicol..

[18] Anderson B., Bell M. (2009). Weather-related mortality: How heat, cold, and heat waves affect mortality in the United States. Epidemiology.

[19] Basu R., Ostro B. (2008). A multicounty analysis identifying the populations vulnerable to mortality associated with high ambient temperature in California. Am. J. Epidemiology.

[20] Kolb S., Radon K., Valois M., Héguy L., Goldberg M. (2007). The short-term influence of weather on daily mortality in congestive heart failure. Arch. Environ. Occup. Health.

[21] Hajat S., Armstrong B., Gouveia N., Wilkinson P. (2005). Mortality displacement of heat-related deaths: A comparison of Delhi, São Paulo, and London. Epidemiology.

[22] Huang C., Barnett A., Wang X., Tong S. (2012). The impact of temperature on years of life lost in Brisbane, Australia. Nature Climate Change.

[23] Baccini M., Kosatsky T., Biggeri A. (2013). Impact of summer heat on urban population mortality in Europe during the 1990s: An evaluation of years of life lost adjusted for harvesting. Plos One.

[24] Gasparrini A., Leone M. (2014). Attributable risk from distributed lag models. BMC Med. Res. Methodol..

[25] Lopez A., Mathers C., Ezzati M., Jamison D., CJL M. (2006). Global Burden of Disease and Risk Factors.

[26] Kenya National Bureau of Statistics (KNBS) (2010). Republic of Kenya Population and Census Survey 2009.

[27] Donev D., Zaletel-Kragelj L., Bjegovic V., Burazeri G. Measuring the Burden of Disease: Disability Adjusted Life Year (DALY). http://www.mf.uni-lj.si/dokumenti/6b695fc9385e3e2ab8fb41ec7d34660d.pdf.

[28] Tank A.M.G.K., Zwiers F.W., Zhang X. (2009). Guidelines on Analysis of Extremes in a Changing Climate in Support of Informed Decisions for Adaptation.

[29] Peng R., Bobb J., Tebaldi C., McDaniel L., Bell M., Dominici F. (2011). Towards a quantitative estimate of future heat wave mortality under global climate change. Environ. Health Perspect..

[30] Meehl G.A., Tebaldi C. (2004). More intense, more frequent, and longer lasting heat waves in the 21st century. Science.

[31] Robinson P.J. (2001). On the definition of a heat wave. J. Appl. Meteorol..

[32] Gasparrini A., Armstrong B. (2011). The impact of heat waves on mortality. Epidemiology.

[33] Rocklöv J., Barnett A.G., Woodward A. (2012). On the estimation of heat-intensity and heat duration effects in time series models of temperature-related mortality in Stockholm, Sweden. Environ. Health.

[34] Gasparrini A., Armstrong B., Kenward M. (2010). Distributed lag non-linear models. Stat. Med..

[35] Basu R. (2009). High ambient temperature and mortality: A review of epidemiologic studies from 2001 to 2008. Environ. Health.

[36] Huang C., Barnett A.G., Wang X., Tong S. (2012). Effects of extreme temperatures on years of life lost for cardiovascular deaths: A time series study in Brisbane, Australia. Circ. Cardiovasc. Qual. Outcomes.

[37] Aragón T., Lichtensztajn D., Katcher B., Reiter R., Katz M. (2008). Calculating expected years of life lost for assessing local ethnic disparities in causes of premature death. BMC Public Health.

[38] Mercer J., Østerud B., Tveita T. (1999). The effect of short-term cold exposure on risk factors for cardiovascular disease. Thromb. Res..

[39] Sun Z. (2010). Cardiovascular responses to cold exposure. Front. Biosci..

[40] Wolf K., Schneider A., Breitner S., von Klot S., Meisinger C., Cyrys J., Hymer H., Wichmann H., Peters A. (2009). Air temperature and the occurrence of myocardial infarction in Augsburg, Germany. Circulation.

[41] Pio A., Kirkwood B.R., Gove S. (2010). Avoiding hypothermia, an intervention to prevent morbidity and mortality from pneumonia in young children. Pediatr. Infect. Dis. J..

[42] Cheng X., Su H. (2010). Effects of climatic temperature stress on cardiovascular diseases. Eur. J. Intern. Med..

[43] Donaldson G., Keatinge W., Saunders R. (2003). Cardiovascular responses to heat stress and their adverse consequences in healthy and vulnerable human populations. Int. J. Hyperth..

[44] Bhaskaran K., Hajat S., Haines A., Herrett E., Wilkinson P., Smeeth L. (2010). Short term effects of temperature on risk of myocardial infarction in England and wales: Time series regression analysis of the myocardial ischaemia national audit project (MINAP) registry. BMJ.

[45] Halonen J., Zanobetti A., Sparrow D., Vokonas P., Schwartz J. (2011). Outdoor temperature is associated with serum HDL and LDL. Environ. Res..

[46] Ren C., O’Neill M., Park S., Sparrow D., Vokonas P., Schwartz J. (2011). Ambient temperature, air pollution, and heart rate variability in an aging population. Am. J. Epidemiol..

[47] Berko J., Ingram D.D., Saha S., Parker J.D. (2014). Deaths Attributed to Heat, Cold, and Other Weather Events in the United States, 2006–2010.

[48] Gasparrini A., Guo Y., Hashizume M., Lavigne E., Zanobetti A., Schwartz J., Tobias A., Tong S., Rocklöv J., Forsberg B. (2015). Mortality risk attributable to high and low ambient temperature: A multi-country study. The Lancet.

[49] Mwangangi A.S., Simiyu C.N. (2014). Analysis of low cost residential housing development for the urban poor: A case study of Kibera and Mathare slums in Nairobi. J. Econom. Sustain. Dev..

[50] Xu Y., Dadvand P., Barrera-Gómez J., Sartini C., Marí-Dell’Olmo M., Borrell C., Medina-Ramón M., Sunyer J., Basagaña X. (2013). Differences on the effect of heat waves on mortality by sociodemographic and urban landscape characteristics. J. Epidemiol. Community Health.

